# Aerosolized Human Extracellular Superoxide Dismutase Prevents Hyperoxia-Induced Lung Injury

**DOI:** 10.1371/journal.pone.0026870

**Published:** 2011-10-26

**Authors:** Chih-Ching Yen, Yi-Wen Lai, Hsiao-Ling Chen, Cheng-Wei Lai, Chien-Yu Lin, Wei Chen, Yu-Ping Kuan, Wu-Huei Hsu, Chuan-Mu Chen

**Affiliations:** 1 Department of Life Sciences, and Agricultural Biotechnology Center, National Chung Hsing University, Taichung, Taiwan; 2 Department of Internal Medicine, China Medical University Hospital, Taichung, Taiwan; 3 Department of Bioresources, Da-Yeh University, Changhwa, Taiwan; 4 Department of Internal Medicine, Chia-Yi Christian Hospital, Chia-Yi, Taiwan; 5 School of Chinese Medicine, China Medical University, Taichung, Taiwan; 6 College of Health Care, China Medical University, Taichung, Taiwan; 7 College of Medicine, China Medical University, Taichung, Taiwan; University of Delhi, India

## Abstract

An important issue in critical care medicine is the identification of ways to protect the lungs from oxygen toxicity and reduce systemic oxidative stress in conditions requiring mechanical ventilation and high levels of oxygen. One way to prevent oxygen toxicity is to augment antioxidant enzyme activity in the respiratory system. The current study investigated the ability of aerosolized extracellular superoxide dismutase (EC-SOD) to protect the lungs from hyperoxic injury. Recombinant human EC-SOD (rhEC-SOD) was produced from a synthetic cassette constructed in the methylotrophic yeast *Pichia pastoris*. Female CD-1 mice were exposed in hyperoxia (FiO2>95%) to induce lung injury. The therapeutic effects of EC-SOD and copper-zinc SOD (CuZn-SOD) via an aerosol delivery system for lung injury and systemic oxidative stress at 24, 48, 72 and 96 h of hyperoxia were measured by bronchoalveolar lavage, wet/dry ratio, lung histology, and 8-oxo-2′-deoxyguanosine (8-oxo-dG) in lung and liver tissues. After exposure to hyperoxia, the wet/dry weight ratio remained stable before day 2 but increased significantly after day 3. The levels of oxidative biomarker 8-oxo-dG in the lung and liver were significantly decreased on day 2 (P<0.01) but the marker in the liver increased abruptly after day 3 of hyperoxia when the mortality increased. Treatment with aerosolized rhEC-SOD increased the survival rate at day 3 under hyperoxia to 95.8%, which was significantly higher than that of the control group (57.1%), albumin treated group (33.3%), and CuZn-SOD treated group (75%). The protective effects of EC-SOD against hyperoxia were further confirmed by reduced lung edema and systemic oxidative stress. Aerosolized EC-SOD protected mice against oxygen toxicity and reduced mortality in a hyperoxic model. The results encourage the use of an aerosol therapy with EC-SOD in intensive care units to reduce oxidative injury in patients with severe hypoxemic respiratory failure, including acute respiratory distress syndrome (ARDS).

## Introduction

Acute lung injury (ALI) and its severe form, acute respiratory distress syndrome (ARDS), are common causes of morbidity and mortality in intensive care units. ALI occurs as a result of direct intra-alveolar injury or indirect injury following systemic inflammation. ALI is characterized by refractory hypoxemia due to widespread alveolar flooding after insult. Currently, the primary management of ALI includes treatment for underlying diseases, adequate hemodynamic support and mechanical ventilation with lung-protective strategies [1]. To maintain adequate tissue oxygenation, higher levels of supplemental oxygen are often required.

In most mammalian species, exposure to hyperoxia can result in lung injury and commonly produces pathological changes similar to those seen in ARDS. Although similar findings have not been reproduced in humans with healthy lungs, most clinicians suspect that oxygen may exacerbate and even cause ALI in critically ill patients [2]. A recent study revealed that even moderate hyperoxia (FiO_2_ = 50%) exacerbates ventilator-induced lung injury (VILI) in a rabbit model [3]. Clinical studies have supported the important role that oxidative stress plays an in the pathogenesis of ALI and other lung diseases, including pulmonary fibrosis, chronic obstructive pulmonary disease, and bronchopulmonary dysplasia [4]–[6].

Oxygen toxicity is mediated by reactive oxygen species (ROS) [7] including superoxide anion (O_2_^−^), hydrogen peroxide (H_2_O_2_), and hydroxyl radicals (⋅OH). ROS can cause lipid peroxidation, oxidation of proteins and DNA damage, which can all induce cellular dysfunction and even cell death [8]. To counteract ROS, a complex network of antioxidants, including enzymatic and non-enzymatic antioxidants, exists in biological systems. Enzymatic antioxidants, which include superoxide dismutase (SOD), catalase, and glutathione peroxidase, provide the first line of defense against ROS.

Superoxide dismutases are a group of isoenzymes that function as key antioxidants in the metabolism of oxygen free radicals. They catalyze the dismutation of superoxide to oxygen (O_2_) and hydrogen peroxide, thereby maintaining a low concentration of the toxic superoxide free radical. Superoxide dismutases are metalloenzymes and exist in three different forms in mammals. Copper-zinc SOD (CuZn-SOD or SOD1) is found in the cytoplasma and nuclei of cells, manganese SOD (Mn-SOD or SOD2) is found in the mitochondria, and Cu/Zn-containing extracellular SOD (EC-SOD or SOD3) is predominantly found in the extracellular matrix of tissues [8], [9].

EC-SOD has been reported to be a multimeric glycoprotein composed of at least four identical 30 kD subunits with heterogeneous affinity for heparin in extracellular matrix (ECM) and cell surfaces [10]. EC-SOD activity has been reported to be markedly increased in the lung compared with other vital organs, such as the liver, kidney, heart, and brain [8]. EC-SOD has been proposed to play an important role in reduction of extracellular oxidative stress resulting from direct exposure of the lung to the external environment [11]. A recent report using Cre-lox technology revealed that an acute 50% reduction of EC-SOD led to a five-fold increase in lung superoxide anions, acute lung injury and 85% mortality within 7 days in the presence of room air [12]. An overexpression of EC-SOD in the airway of transgenic mice attenuates the acute inflammation and protects the lung against hyperoxia [13]. An exposure to 100% oxygen for 72 h can induce proteolysis and depletion of EC-SOD and enhance oxidant/anti-oxidant imbalance in alveolar spaces [14].

heterogeneousA rational strategy for prevention of oxygen toxicity is the augmentation of antioxidant enzyme activity in the respiratory system. Administration of recombinant human CuZn-SOD and Mn-SOD by an aerosol delivery system has been found to protect the lungs against hyperoxic injury [15], [16]. Recombinant human EC-SOD (rhEC-SOD) was expressed and purified in both *E. coli* and the milk of rabbits [17], [18], but no *in vivo* activity studies have been performed. In this study, we produced rhEC-SOD in *Pichia pastoris*, a methylotrophic yeast, and tested the hypothesis that aerosolized rhEC-SOD could protect against oxygen toxicity in a hyperoxic model. We also investigated whether 8-oxo-dG was a useful biomarker for hyperoxic injury. In doing so, we found that rhEC-SOD attenuated hyperoxic lung injury, reduced systemic oxidative stress and increased survival in hyperoxia.

## Materials and Methods

### Production of human EC-SOD in *Pichia pastoris*

**Figure 1:**
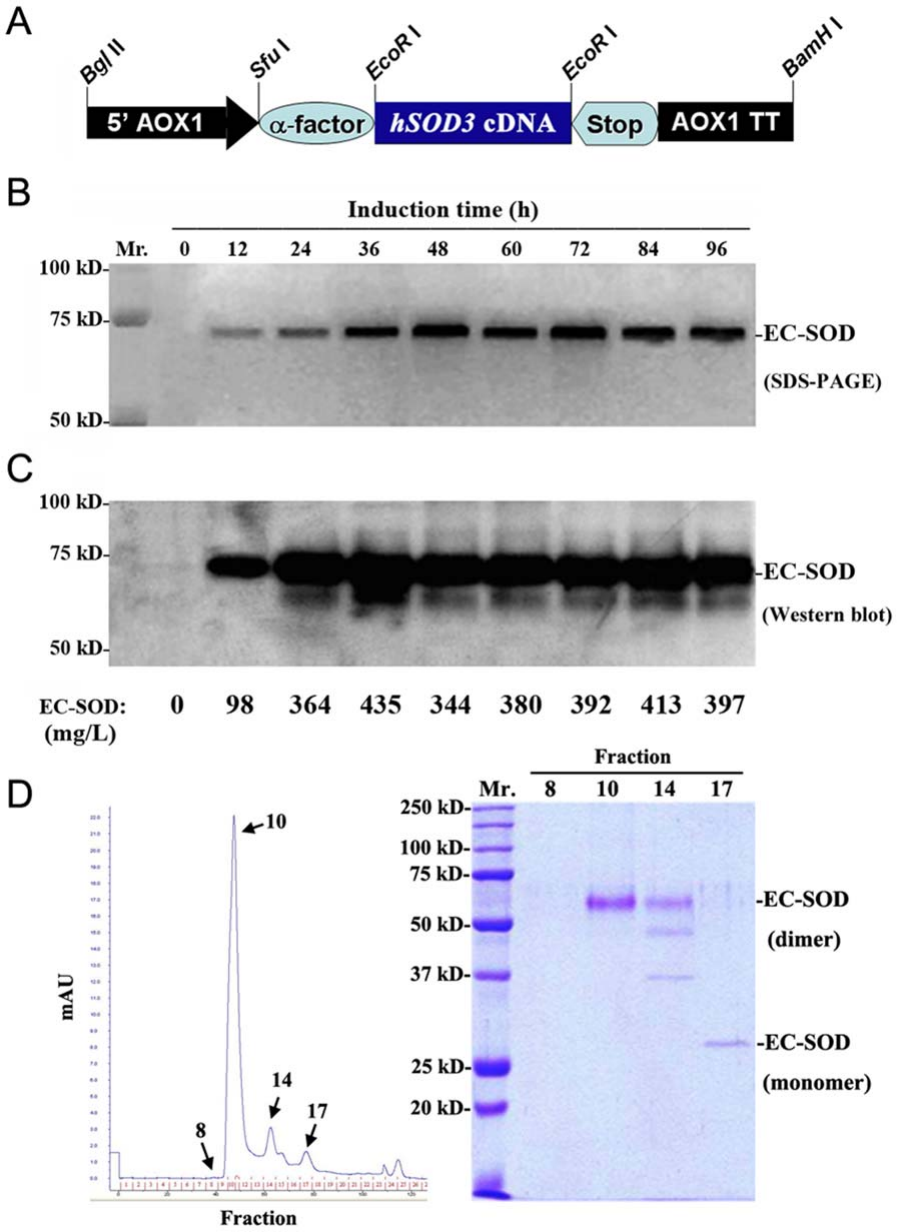
Production and secretion of rhEC-SOD in *Pichia pastoris*. **A.** Structure of the *P. pastoris* α-factor/human-EC-SOD secretion cassette. The hSOD3 cDNA fragment was cloned into pPICZαA vector The *AOX1* gene can easily be induced by methanol. **B**. Representative SDS-PAGE analysis of time course of rhEC-SOD expression and secretion in the yeast cultures after methanol induction. **C.** Western blot analysis of secreted rhEC-SOD with a mouse anti-human EC-SOD monoclonal antibody. The amount of rhEC-SOD was determined quantitatively by ELISA. Results are representative of three experiments. **D.** The purification of rhEC-SOD by FPLC system. The purity and molecular weight of purified rhEC-SOD (fraction No. 10) was checked by 12% SDS-PAGE.

The construction, screening, and production of human EC-SOD have been described in detail elsewhere [9]. Briefly, the *hSOD3* cDNA fragment was amplified by PCR and cloned into pPICZαA yeast expression vector (Figure 1A). After electroporation-stimulated transformation into *P. pastoris*, selected the pPICZαA-hSOD3-transformed colonies with high levels of Zeocin. Recombinant hEC-SOD protein was produced and secreted into the culture medium under the induction of methanol. Three liters of culture medium were concentrated by stirred-cell ultrafiltration (YM-10, Amicon, Danvers, MA). The precipitate was resuspended in a 5 mM Tris buffer (pH 7.4) containing 50 mM NaCl and dialyzed against the same buffer. The desalted fractions were separated and purified by a fast protein liquid chromatography (FPLC) system (AKTA purifier 10, Amersham Pharmacia Biotech., Arlington Heights, IL) [9]. The activity unit of rhEC-SOD antioxidant was assayed with a water-soluble tetrazolium salt (WST-1) kit (Dojindo Molecular Technologies, Inc., Rockville, MD) as described [9].

### Experimental animals

Six- to eight-week-old female CD-1 strain mice weighing 20–30 g were purchased from BioLASCO Taiwan, INC. The mice were housed in an SPF-grade animal facility under a 12-h light/12-h dark cycle with a constant temperature (25±1°C). Mice were provided with food and water *ad libitum* throughout the experiment. This study was conducted according to institutional guidelines and approved by the Institutional Animal Care and Utilization Committee of National Chung-Hsing University, Taiwan (IACUC No. 96-52).

### Oxygen exposure and aerosol delivery system

Mice were exposed to hyperoxic conditions (FiO_2_ >95%) in a 36×20×20-cm plexiglass chamber with a hole (2 cm in diameter) to allow the continuous flow of 100% oxygen (1 L/min) and aerosol into the chamber. Oxygen level was very stable (97–99%, showed in Figure S1) when monitored every hour in light cycle of housing with an oxygen analyzer (MiniOX I, MSA Canada, Inc., Canada). Two ultrasonic nebulizers (SUMO V15, Taiwan) were used for the aerosolization of the study drugs, and they were connected to the oxygen delivery system.

### Experimental protocol

The first aim of the experiments was to demonstrate the time course of lung injury and oxidative stress by examining the effects of hyperoxic conditions at 24, 48, 72, and 96 h. At each time point, six mice in each group were sacrificed for the following studies including bronchoalveolar lavage, wet/dry ratio, lung histology, and 8-oxo-dG determination in lung and liver tissues (n = 6 in each group). The second aim of the experiments was to test the hypothesis that aerosolized EC-SOD and CuZn-SOD could reduce systemic oxidative stress and lung injury after 96 h in hyperoxic conditions. The mice were divided into three groups: 1) SOD1 inhalation (10,000 units/day, bovine CuZn-SOD from Sigma, USA), 2) SOD3 inhalation (10,000 units/day, rhEC-SOD produced by this study), 3) albumin inhalation (890 µg/mL in PBS, equal to the SOD3 concentration, as a non-SOD control), and 4) PBS inhalation (as a control). At least six mice were recruited in each group. SODs or albumin solved in PBS 70 mL or PBS 70 mL alone was delivered by this aerosol system in 8 h per day. About 36% of the weight of solutions was left in the system 8 hours after aerosolization. The health of mice was monitored daily throughout the experimental period. The survival rate was also monitored daily, and at least six mice from each group were sacrificed at 96 h for the experiments.

### Bronchoalveolar lavage

The trachea was exposed with a midline incision and cannulated with a modified 21-gauge needle. After preliminary test, bronchoalveolar lavage (BAL) was performed four times with 2.5 mL PBS per time. At least 1 mL was recovered after each lavage. The BAL fluid was mixed and centrifuged at 500× g for 10 min at 4°C. Cell pellets were resuspended in 1 mL PBS, and cell counts were performed [16]. The supernatant was submitted to total protein analysis using a bicinchoninic acid (BCA) protein assay (Pierce, Rockford, IL).

### Lung wet/dry weight ratio

After euthanasia of the mice, the lungs were surgically dissected away from the heart, trachea, and main bronchi. Each lung was blotted dry, weighed, and dried to a constant weight by placing the lung specimen in an oven at 70°C for 48 h. The ratio of the wet lung to the dry lung was calculated to assess lung edema.

### Lung pathology and immunohistochemical staining of EC-SOD

After sacrificing the mice, the left lobes of the lung were dissected and inflated with 0.6 mL of 10% formalin for histological study. Paraffin sections prepared from the lungs were stained with hematoxylin and eosin for evaluation. We assessed the degree of alveolar congestion, hemorrhage, leukocyte infiltration, and the thickness of the alveolar wall [19]. For immunohistochemical (IHC) staining of EC-SOD, the lung tissue was fixed with paraformaldehyde and embedded in O.C.T. compound (Tissue-Tek^R^; Sakura, Japan), then frozen and microdissected for IHC analysis [20]. Briefly 5 µm section placed on slides were incubated with rabbit anti-rhEC-SOD polycolonal first antibody (1∶200 dilution; EMD Millipore, Billerica, MA) and biotin-labeled anti-rabbit IgG antibody (1∶2,000 dilution; Abcam, Cambridge, MA). The Vectastain ABC kit (Vector Laboratories, Burlingame, CA) was used for rhEC-SOD staining.

### 8-oxo-dG analysis of lung and liver tissues

Mice were sacrificed to get lung and liver tissues for quantitative determination of 8-oxo-dG. Deoxyribonucleic acid extractions were performed as previously described [21], and DNA digestions were performed by following the Dig-1 protocol as described previously [22]. Artificial oxidation was minimized by the NaI/2-propanol method. Isotope-dilution liquid chromatography-tandem mass spectrometry (LC-MS/MS) with on-line solid-phase extraction was used to measure 8-oxo-dG as previously described [23]. After the addition of ^15^N-labeled 8-oxo-dG as an internal standard, the samples were analyzed within 10 min.

### Data analysis

All experimental results were expressed as mean ± SE. The Levene test was used to determine whether values were normally distributed. After this assessment, either the Mann-Whitney U test or the Student's *t* test was applied (StatView, Abacus). Differences with p<0.05 (*) or p<0.01 (**) were considered to be statistically significant.

## Results

### Recombinant human EC-SOD expression and purification

To produce bioactive rhEC-SOD antioxidant, a synthetic secretion cassette for human EC-SOD in yeast vector was constructed and transformed into *P. pastoris*. Twenty-four hours after induction, the amount of rhEC-SOD in the yeast culture was significantly increased as shown in the SDS-PAGE (Figure 1B). The secreted dimeric rhEC-SOD (70 kD) in the culture medium was further assayed by western blot and ELISA (Figure 1C). After purification with FPLC, the pure dimeric rhEC-SOD was collected from fraction No. 10 (Figure 1D) with a specific activity of 251.72 U/mg. A total activity of 10,000 units/mL of rhEC-SOD was prepared for the experiments.

### Effect of oxidative stress on lung injury and mortality after hyperoxia

**Figure 2:**
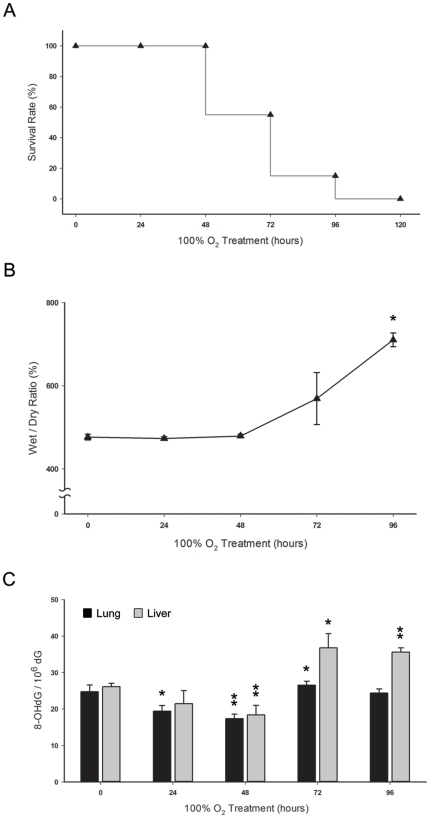
Effects of oxidative stress on lung injury and survival in hyperoxia (FiO_2_ >95%). **A.** The survival of untreated mice after exposure to hyperoxic conditions (n = 24). **B.** Pulmonary edema was measured based on the wet/dry weight ratio of the lungs (n = 6 in each group). **C.** Time course of oxidative stress in lung and liver tissues after exposure to hyperoxic conditions (n = 6 in each group). The oxidative marker 8-oxo-dG was determined with LC-MS/MS with on-line SPE. Values are expressed as the amount of 8-oxo-dG per 10^6^ dG.

When CD-1 mice were exposed to hyperoxic conditions (FiO_2_ >95%), 100% of the mice died by day 5 (Figure 2A). The wet/dry weight ratio, which was used as a marker of lung edema, remained stable before day 2 but increased significantly after day 3 and day 4 (P<0.05) (Figure 2B). The levels of 8-oxo-dG (Figure 2C) in the lungs decreased significantly at day 2 (P<0.01) and increased gradually after day 3. Similarly, the level of 8-oxo-dG in liver tissue, which has been proposed as a measure of systemic oxidative stress, decreased before day 2 and increased at day 3 (P<0.05). The abrupt elevation of 8-oxo-dG in the liver tissue on day 3 was concurrent with the development of lung edema and a decline in the survival curve.

### Aerosolized rhEC-SOD increased survival from hyperoxia

**Figure 3:**
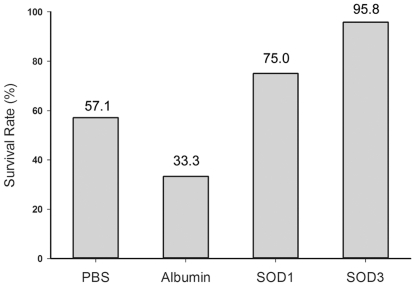
Effects of aerosolized CuZn-SOD and rhEC-SOD on survival after 72 h of hyperoxia. Phosphate-buffered saline (PBS) inhalation was used as the hyperoxia-induced lung injury control group. The three treatment groups were Albumin, SOD1 (CuZn-SOD), and SOD3 (rhEC-SOD) (n = 24 in each group). The albumin treated group was added as a non-SOD inhalation control group.

The survival rate of the PBS inhalation group was 57.1% after 72 h of hyperoxia (Figure 3). Treatment with aerosolized rhEC-SOD increased the survival rate to 95.8% at 72 h after hyperoxia exposure, which was significantly higher than that in the control (57.1%, P<0.01), albumin (33.3%, P<0.01), and CuZn-SOD (75.0%, P<0.05) groups. Aerosolized CuZn-SOD seemed to increase the survival, but the results were not significant compared with the control group (P = 0.098). Mice that survived beyond day 3 in the PBS group were clearly impaired and had limited movement. In contrast, the mice in the rhEC-SOD group demonstrated remarkable tolerance to hyperoxia (as shown in the Video S1).

### Aerosolized rhEC-SOD and CuZn-SOD reduced hyperoxic lung edema

**Figure 4:**
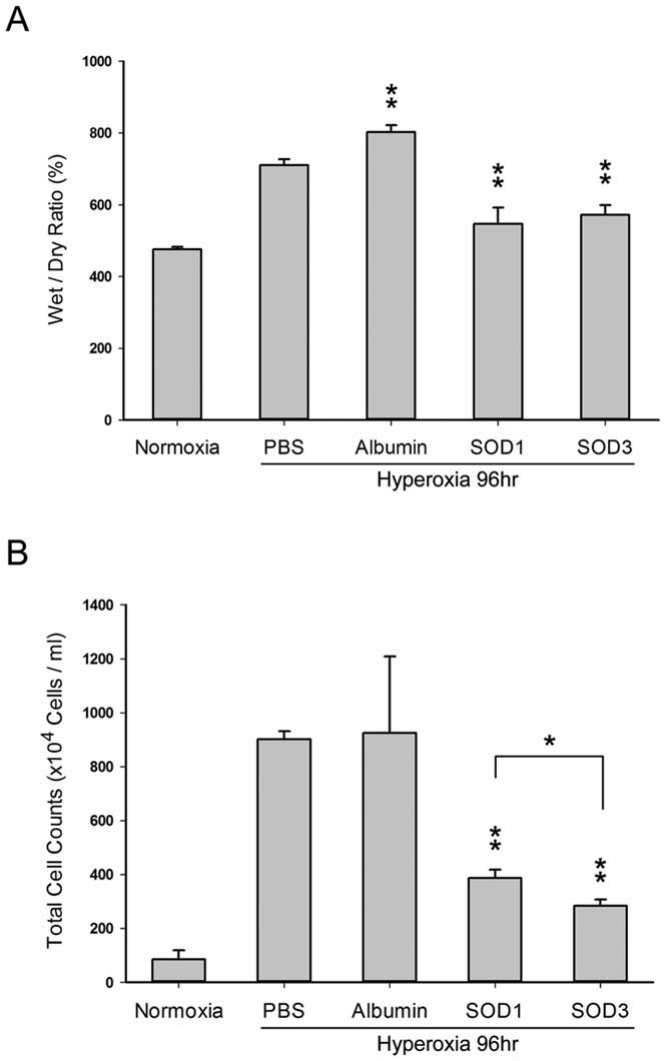
Effects of aerosolized CuZn-SOD and rhEC-SOD on lung injury after 96 h of hyperoxia. **A.** Pulmonary edema demonstrated by the wet/dry weight ratio of the lungs (n = 6 in each group). **B.** Total cell count in BAL fluid (n = 6 in each group; * P<0.05; ** P<0.01).

The wet/dry weight ratios of the lungs from the SOD-treated groups (Figure 4A) were significantly lower than the ratios in the PBS control group (P<0.01), but there was no significant difference between the CuZn-SOD and rhEC-SOD groups. The total cell count in the BAL fluid, a marker of lung injury, was also significantly lower in the SOD-treated groups compared with the PBS or albumin control group (P<0.01; Figure 4B). In addition, the total cell count in the BAL fluid of the rhEC-SOD group was even lower than that in the CuZn-SOD group (P<0.05).

### Protective effect of rhEC-SOD against hyperoxic lung injury was greater than the effect of CuZn-SOD

**Figure 5:**
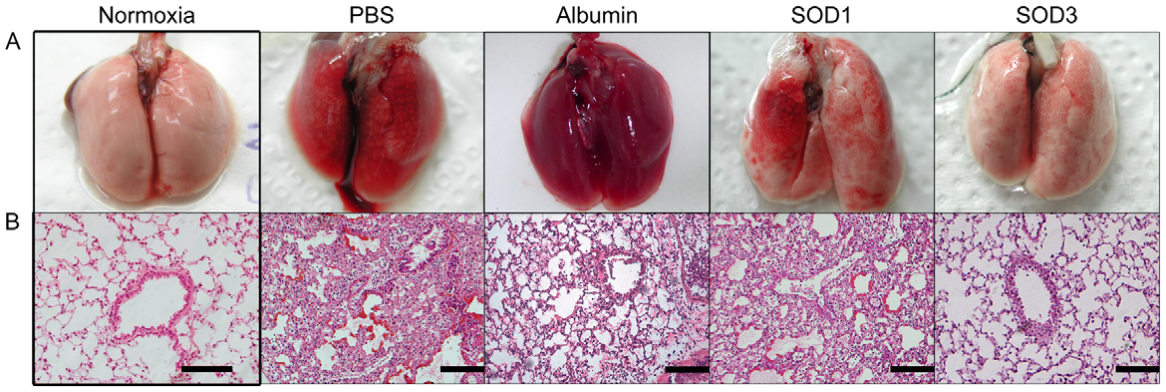
Effects of aerosolized CuZn-SOD and rhEC-SOD on lung morphology after 96 h of hyperoxia. Effects are shown as overview of full lung organ (**A**), and histological lung section with H&E staining (**B**). The control group of mice fed in normal oxygen condition (Normoxia) was shown in left panel. Following by inhalations of PBS, Albumin, SOD1 (CuZn-SOD), and SOD3 (rhEC-SOD) treated groups (n = 6 in each group) under the hyperoxia condition (FiO_2_>95%). The scale bars represent 500 µm.

**Figure 6:**
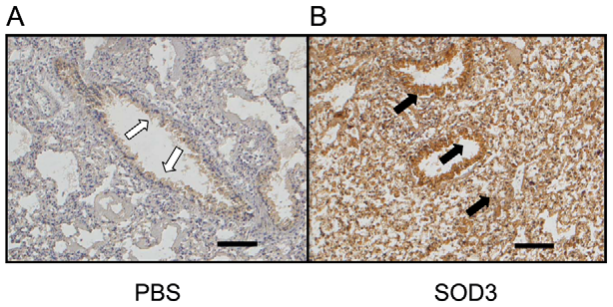
The distribution of aerosolized rhEC-SOD in lung tissue by immunohistochemical (IHC) staining after 96 h of hyperoxia. **A.** The image representative the PBS inhalation control group. Only a few of endogenous EC-SOD can be detected in the bronchial epithelia as indicated by open arrows. **B.** The image representative the rhEC-SOD (SOD3) aerosolized group. The exogenous rhEC-SOD can be strongly detected in the bronchial epithelia, alveoli and lung parenchyma as indicated by solid black arrows. Scale bar = 100 µm.

To further confirm the protective effect of rhEC-SOD, histopathological examination of lungs was performed after 96 h of hyperoxia. Pulmonary edema and alveolar infiltration of neutrophils were evident in the PBS, albumin, and CuZn-SOD groups (Figure 5). Mice from the group that received aerosolized rhEC-SOD exhibited less neutrophil infiltration and lung edema (Figure 5A and 5B). Immunohistochemical staining of EC-SOD was performed to assess the efficiency of this aerosol system and the distribution of EC-SOD in lung after aerosolization. In the EC-SOD treatment group, EC-SOD was expressed diffusely in lungs, including epithelium of airway, alveolar space, and even interstitium (Figure 6B). Because of the cross reaction of the polyclonal antibodies with endogenous EC-SOD, EC-SOD was found in the epithelium of bronchi and bronchiole in the PBS control group (Figure 6A).

### Protective effect of rhEC-SOD against systemic oxidative stress was greater than that of CuZn-SOD

**Figure 7:**
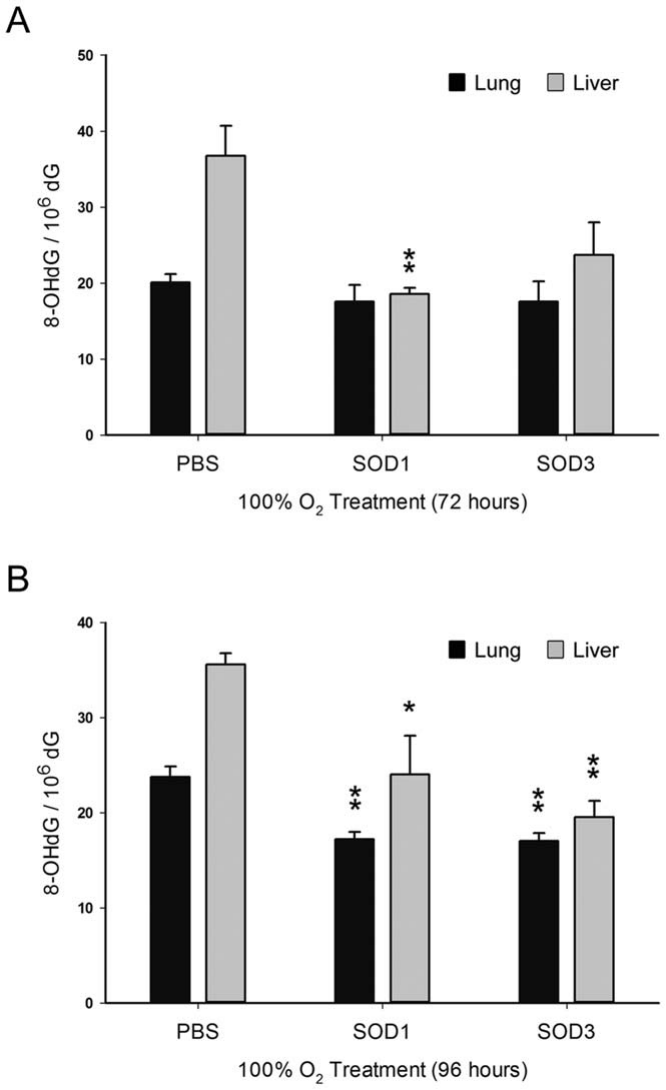
Effects of aerosolized CuZn-SOD and rhEC-SOD on oxidative stress in lung and liver tissues after 72 h and 96 h of hyperoxia. The levels of the oxidative stress biomarker 8-oxo-dG in lung and liver tissues, measured with LC-MS/MS with on-line SPE, after 72 h (**A**) and 96 h (**B**) hyperoxia treatments. Values are expressed as the amount of 8-oxo-dG per 10^6^ dG (PBS was used as the control group; n = 6 in each group; * P<0.05; ** P<0.01).

To test the hypothesis that aerosolized rhEC-SOD reduced systemic oxidative stress and increased survival following hyperoxia, we measured the levels of 8-oxo-dG in lung and liver tissues after 72 h and 96 h in hyperoxic conditions. The levels of 8-oxo-dG in the lung tissues at day 3 (Figure 7A) and day 4 (Figure 7B) were reduced by treatment with aerosolized rhEC-SOD and CuZn-SOD. The difference between the protective effects in the rhEC-SOD (P<0.01) and CuZn-SOD (P<0.05) groups was shown in the liver tissues, but did not reflect the equivalent reduction of 8-oxo-dG in the lung tissues under 96 h hyperoxic condition (Figure 7B).

## Discussion

There were two new findings in this study. First, aerosolized human EC-SOD administration in mice protected against acute lung injury and systemic oxidative stress caused by hyperoxia (FiO_2_>95%). Extracellular superoxide dismutase aerosol therapy reduced the severity of lung injury, which was demonstrated by the wet/dry weight ratio and histopathology, as well as the systemic oxidative stress, which was demonstrated by 8-oxo-dG in the liver. In addition, EC-SOD reduced the mortality rate. The protective effect of rhEC-SOD against oxygen toxicity was greater than the effect of bovine CuZn-SOD in this animal model. Second, we found that 8-oxo-dG could be used as an oxidative biomarker in a hyperoxic model. Systemic oxidative stress, demonstrated by 8-oxo-dG in the nuclei of liver tissue, was concurrent with the development of pulmonary edema, the decline in the survival curve and the protective effects of aerosol SODs.

Numerous studies have shown that oxidants are important mediators of ALI, and augmentation of antioxidant enzymes is protective in animal models of ALI. SODs, especially EC-SOD, have been found to play an important role in the pathogenesis of ALI. EC-SOD knockout mice have exaggerated inflammation in response to hyperoxia [24] and lipopolysaccharide (LPS) challenge [25]. On the other side, an overexpression of EC-SOD in the airway of transgenic mice attenuates ALI and protects the lung against hyperoxia [13]. The role of EC-SOD was further confirmed by the report that an acute reduction of EC-SOD in adult mice led to ALI and mortality even in room air [12]. In the same report, intraperitoneal injection of the SOD mimetic MnTBAP and intranasal administration of CuZnSOD-containing polyketal microparticles reduced the lung injury and mortality [12]. Mn-SOD knockout mice usually die within ten days after birth, whereas transgenic mice overexpressing human CuZn-SOD or Mn-SOD show increased tolerance to oxygen toxicity [26]–[28]. One clinical study revealed that subcutaneous injection of bovine CuZn-SOD may be helpful in reducing bronchopulmonary dysplasia in preterm infants [7]. To the best of our knowledge, this study is the first report of the protective effects of aerosolized hEC-SOD in hyperoxia.

Superoxide anion plays a key role in oxygen toxicity. Superoxide can undergo SOD dismutation to H_2_O_2_, which is comparatively stable, or react with nitric oxide (NO) to form peroxynitrite (ONOO^−^). Superoxide anions can even participate in Fenton reactions to produce hydroxyl radicals (OH^−^). The relatively insufficiency of EC-SOD caused by overproduction of superoxide or proteolysis of EC-SOD induced by hyperoxia [14] has been shown to direct the reactions towards the formation of ONOO^−^ and OH^−^, which are highly reactive products and important mediators of hyperoxia-induced lung injury [29]. The activities of glutathione peroxidase and catalase, which catalyze H_2_O_2_ to H_2_O, have been shown to increase after continuous exposure to hyperoxic conditions [30]. An exogenous EC-SOD supplement like the aerosol therapy in this experiment could prevent accumulation of superoxide anions and its associated oxidants and increase oxygen tolerance in hyperoxia. Another important protective effect of EC-SOD involves its ability to inhibit inflammation [13], [31]. ROS can induce fragmentation of several components of ECM (collagen, hyaluronan, and syndecan-1, a heparan sulfate proteoglycan) and then the following chemotaxis of neutrophils and inflammation. One of the anti-inflammatory mechanisms of EC-SOD is by the binding to the components of ECM and preventing their degradation [32]–[34]. In ischemia and peritonitis models, EC-SOD gene transfer reduces inflammatory cell migration by reducing the expression of adhesion molecules and proinflammatory cytokines [31].

The anti-inflammatory effect at the cell surface and the chemical properties of EC-SOD may in part be why aerosol therapy with EC-SOD is more effective than treatment with CuZn-SOD in protection against oxygen toxicity. Superoxide produced in the mitochondria or released extracellularly by activated neutrophils is important for the pathogenesis of hyperoxia-induced lung injury, while the role of cytoplasmic superoxide in oxygen toxicity is limited [29]. The anti-oxidant effect of EC-SOD occurs at the surface of alveolar and endothelial cells. EC-SOD prevents the cells from absorbing free radicals, even into the lung parenchyma. Such an “antioxidant screen” outside of alveolar cells could reduce the cell damage as much as possible. EC-SOD protein has been shown to be very stable and displays marked resistance to high temperature [9]; thus, it was suitable for aerosol therapy.

The delivery of aerosol drugs to the respiratory tract has some advantages, including rapid onset, smaller required doses, and a local pulmonary effect with fewer systemic side effects; additionally, it is painless and relatively convenient. In this study, we proved the fact that EC-SOD can be aerosolized effectively and then be absorbed into alveoli and lung parenchyma by IHC staining. The significant higher survival rate of EC-SOD treatment group than that of PBS control, albumin, and CuZn-SOD treatment groups revealed the protective effect against oxygen toxicity of aerosolized EC-SOD. Total cell counts in BAL fluid [35] and pathology of lung revealed that EC-SOD is more effective than CuZn-SOD for reducing lung injury, but the same effect could not be demonstrated by the wet/dry ratio of lung. The aerosolized proteins deposited in lungs are equal in two treatment groups may account for the insignificant difference of wet/dry between EC-SOD and CuZn-SOD groups. The results imply that aerosol therapy may be an effective route of administration of EC-SOD for protection against oxygen toxicity when high oxygen concentration is needed in critically ill patients.

The use of biomarkers for oxidative stress may provide early detection and further evaluation of oxidative damage. Several biomarkers of oxidized lipids, proteins and DNA have been found. 8-OHdG was recently demonstrated as a useful biomarker of oxidative stress in various tissues [36] and even the marker in urine is significantly correlated with the outcome of critically septic patients [37]. When anti-oxidant systems are overwhelmed by external oxidative stress, DNA can be damaged by ROS and then strand breaks and modifications of various bases can occur. The 8-OHdG adduct and its tautomer, 8-oxo-dG, can easily formed in large quantities. The 8-OHdG adduct can mismatch with adenine instead of cytosine, which could cause GC-to-TA transversion and lead to point mutations. The 8-OHdG adduct is cytotoxic by itself because it can induce apoptosis through downregulation of bcl-2, an antiapoptotic protein [38], [39].

In this study, 8-oxo-dG in the lung tissue initially decreased before it increased at day 3. A similar pattern was observed in liver tissue, but there was a more dramatic elevation at day 3. The time course of 8-oxo-dG in liver tissue was compatible with the development of lung edema and the decline in the survival curve. This trend implies that the antioxidant systems compensated well at first, but prolonged oxygen exposure resulted in an elevation of systemic oxidative stress. The levels of 8-oxo-dG were consistent with the protective effect of the SODs. The initial decrease of 8-oxo-dG could have been caused by increased activity of antioxidants and/or activation of the DNA repair system or 8-oxoquanine glycosylase 1 (OGG1). The later decompensation could be the result of the proteolysis of antioxidant enzymes and/or shutdown of the DNA repair systems. Further studies are necessary to determine the mechanism.

Transgenesis using *P. pastoris* as a bioreactor to produce functional protein therapeutic drugs is a promising direction in biotechnology and medicine. *Pichia pastoris* is uniquely suited for expressing transgenic proteins because of its ability to synthesize large amounts of recombinant proteins. In addition, its glycosylation abilities are very similar to those of animal cells. The recombinant proteins made by *P. pastoris* are unlikely to induce immune reactions if they are injected into the bloodstream [40]. Aerosolized human CuZn-SOD in a sheep model and bovine CuZn-SOD for preterm infants were found to be safe throughout the course of treatment [7], [41]. No side effects were found in this aerosolized EC-SOD study, but this preparation will require further investigations before clinical use.

In conclusion, we have successfully expressed human EC-SOD in *P. pastoris* cells. Aerosolized recombinant hEC-SOD protected against oxygen toxicity and reduced mortality in a mouse hyperoxic model. The results of this study are encouraging, and aerosol therapy with rhEC-SOD may permit patients with severe hypoxemic respiratory failure, including ARDS, to receive high levels of oxygen with less oxidative injury.

## Supporting Information

Figure S1 The plot of oxygen levels in hyperoxia chambers measured by oxygen analyzer (MiniOX I, MSA Canada, Inc., Canada) every hour during the housing of light cycle (6:00 am–18:00 pm) for four days.
(JPG)

Video S1 The video of the health condition of mice behavior observed during hyperoxia experiment. Mice that survived beyond day 3 in the Albumin control group were clearly impaired and had limited movement (right panel). In contrast, the mice in the rhEC-SOD group demonstrated remarkable tolerance to hyperoxia (left panel).
(MOV)
