# Supplementary figures and images for: Aerosolized Human Extracellular Superoxide Dismutase Prevents Hyperoxia-Induced Lung Injury
Source: PLoS One. 2011 Oct 26;6(10):e26870. doi: 10.1371/journal.pone.0026870 (PMC3202580; doi:10.1371/journal.pone.0026870)

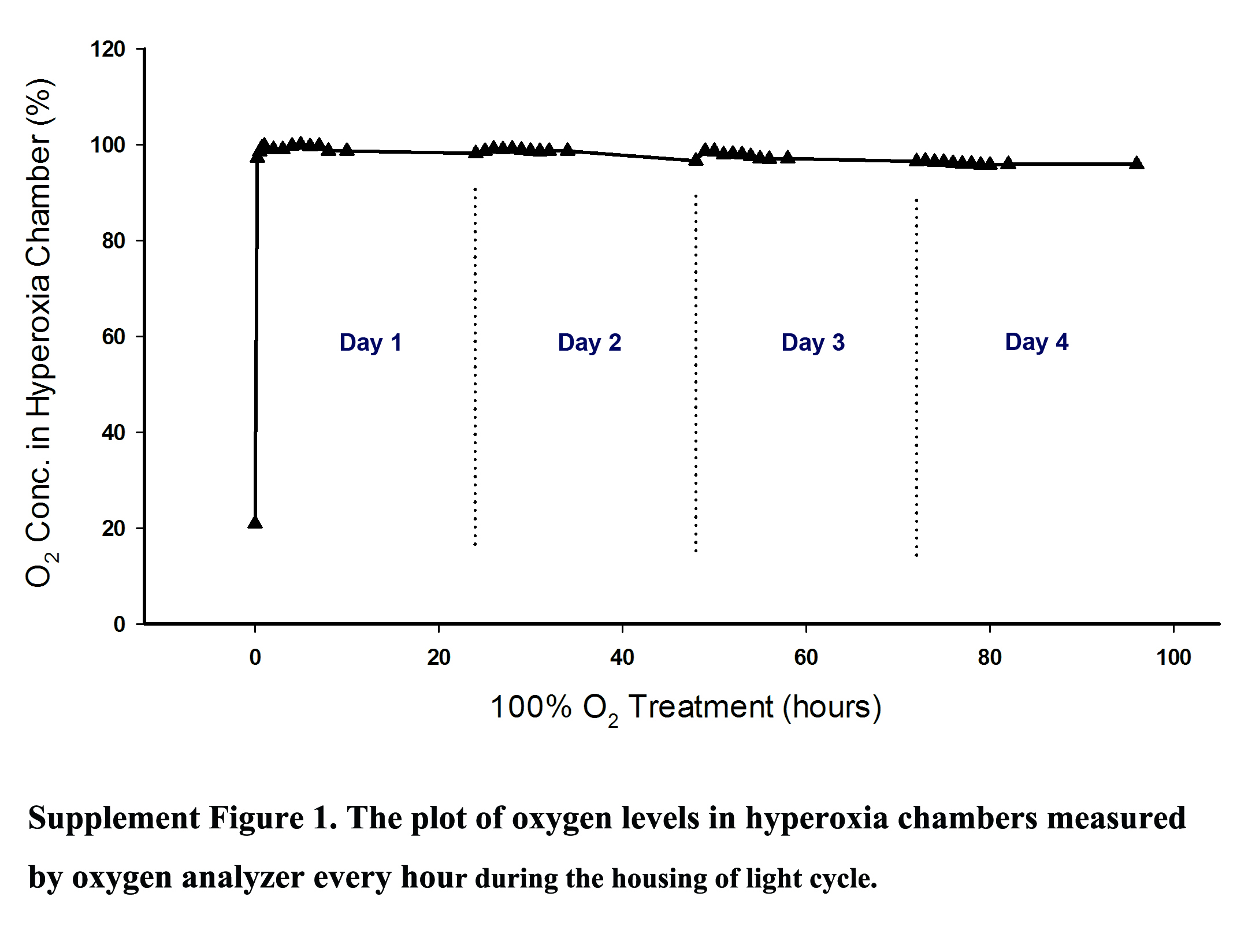

Supplement: Figure S1 — The plot of oxygen levels in hyperoxia chambers measured by oxygen analyzer (MiniOX I, MSA Canada, Inc., Canada) every hour during the housing of light cycle (6:00 am–18:00 pm) for four days. (JPG) [file pone.0026870.s001.jpg]
